# A mapping of facilitators and barriers to evidence-based management in health systems: a scoping review study

**DOI:** 10.1186/s13643-021-01595-8

**Published:** 2021-01-30

**Authors:** Tahereh Shafaghat, Mohammad Hasan Imani Nasab, Mohammad Amin Bahrami, Zahra Kavosi, Mahsa Roozrokh Arshadi Montazer, Mohammad Kazem Rahimi Zarchi, Peivand Bastani

**Affiliations:** 1 Student Research Committee, School of Management and Medical Informatics, Shiraz University of Medical Sciences, Shiraz, Iran; 2 Social Determinants of Health Research Center, Lorestan University of Medical Sciences, Khorramabad, Iran; 3 Health Human Resources Research Center, School of Management and Medical Informatics, Shiraz University of Medical Sciences, Shiraz, Iran; 4 Health Policy and Management Research Center, Department of Health Care Management, School of Public Health, Shahid Sadoughi University of Medical Sciences, Yazd, Iran

**Keywords:** Evidence-based decision-making, Facilitators, Barriers, Health system

## Abstract

**Background:**

Healthcare settings are complex, and the decision-making process is usually complicated, too. Precise use of best evidence from different sources for increasing the desired outcomes is the result of EBM. Therefore, this study aimed to map the potential facilitators and barriers to EBM in health systems to help the healthcare managers to better implement EBM in their organizations.

**Methods:**

The present study was a scoping review (SR) conducted in 2020 based on the integration of the frameworks presented by Arksey and O’Malley (2005) and Levac et al. (2010) considering the Joanna Briggs Institute guideline (2015). These frameworks consist of 6 steps. After finalizing the search strategy, 7 databases were searched, and the PRISMA-ScR was used to manage the retrieval and inclusion of the evidence. Microsoft Excel 2013 was used to extract the data, and the graphic description was presented. The summative analysis approach was used applying MAXQDA10.

**Results:**

According to the systematic search, 4815 studies were retrieved after eliminating duplicates and unrelated articles, 49 articles remained to extract EBM facilitators and barriers. Six main aspects attitude toward EBM, external factors, contextual factors, resources, policies and procedures, and research capacity and data availability were summarized as EBM facilitators. The barriers to EBM were similarly summarized as attitude toward EBM, external factors, contextual factors, policies and procedures, limited resources, and research capacity and data availability. The streamgraphs describe that the international attention to the sub-aspects of facilitators and barriers of EBM has been increased since 2011.

**Conclusions:**

The importance of decision-making regarding complex health systems, especially in terms of resource constraints and uncertainty conditions, requires EBM in the health system as much as possible. Identifying the factors that facilitate the use of evidence, as well as its barriers to management and decision-making in the organization, can play an important role in making systematic and reliable decisions that can be defended by the officials and ultimately lead to greater savings in organization resources and prevent them from being wasted.

**Supplementary Information:**

The online version contains supplementary material available at 10.1186/s13643-021-01595-8.

## Background

In the last decade of the twentieth century, evidence-based medicine was introduced, which is defined as “the conscientious, explicit, and judicious use of current best evidence in making decisions about patient care” [1]. Then, the concept of evidence use in other areas such as management was proposed. Data, information, or evidence would be wealth if they have been used for informed decisions [2]. “Evidence-based management (EBM) is about making decisions through the conscientious, explicit, and judicious use of the best available evidence from multiple sources by asking, acquiring, appraising, aggregating, applying, and assessing to increase the likelihood of a favorable outcome” [3]. Decision-making is the core of managerial tasks, so it can be said that evidence-based decision-making (EBDM) is a subset of EBM.

Healthcare settings are complex; consequently, the types of decisions that must be made are usually complicated too. Often, decisions are based on incomplete and outdated information and personal experiences [4]. Therefore, using evidence in the decision-making process can lead to improving the quality of managerial decisions [5]. Managers should make effective and efficient decisions that lead to better productivity of the organization [1], and the accurate and precise use of best evidence from different sources for increasing the outcomes is the result of EBM [6].

Although some studies have shown that health leaders have a generally positive attitude toward EBM [1], it was applied less than evidence-based medicine in health organizations, so far. Managers do not desire in applying evidence because of existing different barriers [7], and they cannot overcome these barriers and provide the facilitators to better implementation of EBM in their organization until they know and recognize all the possible EBM barriers and facilitators [8, 9].

Recognizing the facilitators and barriers of EBM is necessary to develop this approach and implement it by the health care managers [10]. However, several studies were performed to identify facilitators and barriers to EBM or EBDM in healthcare organizations; they only focused on some aspects of just one or two of these factors and did not present a comprehensive and complete set or framework for them [11–16]. Therefore, providing a complete map of the EBM facilitators and barriers in health systems can provide a comprehensive view that can help prioritize future efforts and promote the implementation of EBM in the health systems [17]. Hence, the main purpose of this study was to develop a map of the potential facilitators and barriers to EBM in health systems. So, we decided to map the EBM facilitators and barriers in health systems using scoping review because of the broad nature of scoping reviews that make them particularly useful for bringing together evidence from disparate or heterogeneous sources and presenting a comprehensive set or framework for desired factors and conditions [18].

## Methods

This was a scoping review conducted in 2020. In order to design the study, the Joanna Briggs Institute’s protocol (2015) was applied, and the integration based on the frameworks presented by Arksey and O’Malley (2005) [18] and Levac et al. (2010) [19] was used. This piece of the manual, as we said, has compared the proposed stages as a framework of scoping review by Arksey and O’Malley [18] and the enhancements suggested by Levac et al. [19]. These frameworks consist of 6 steps. We have tried to compare and integrate these two approaches for a better illustration of mapping the evidences. Also, the PRISMA-ScR was used as a checklist to report this scoping review (see supplementary files 1). The detailed methodology of the scoping review is indicated as follows:

### Selecting the research question

In this step according to the Joanna Briggs Institute manual for scoping reviews (2015), the main research question was defined as “what are the EBM/EBDM facilitators and barriers in health systems/organization?” As the nature of the scoping review’s question is iterative, the specific questions were made as follows:

- What are the facilitators or enablers that help health systems decide according to the evidence?
- What are the barriers or limitations to evidence-based decision-making or evidence-based management in health systems?

In this regard, Levac et al. [19] have enhanced the “identifying the research question” to “clarifying and linking the purpose and research question,” so after defining the research question, the link between the purpose and the research question was clarified.

Furthermore, the scoping review question guides and directs the development of the specific inclusion criteria for the scoping review. The clarity in the review question assists to develop the protocol, facilitate effectiveness in the literature search, and provide a clear structure for the development of the scoping review report. As with the title, the question should incorporate the PCC elements (population, concept, and context) [19]. In this study, the population (P) included all the articles considering the facilitators or enablers of the EBM in health systems and those regarding the barriers, obstacles, or limitations of applying EBM in health systems. The concept (C) was the EBM in health systems, and the context (C) was all the health organizations, health care centers, and health systems that need evidence to behave and decide.

### Searching for related studies

In this step, the authors have searched the 7 main databases including Cochrane, ISI web of science, PubMed, Scopus, Science Direct, ProQuest, and EMBASE applying related keywords. The search duration was defined from January 01, 2000, up to August 25, 2020. Table 1 shows the finalized search strategy of the scoping review. According to Levac et al. [19] in this step, the feasibility and comprehensiveness of the scoping review were considered, and the seven pre-stated databases were finalized to be searched.

**Table 1:** The search strategy of the study

Databases: Cochrane, ISI web of science, PubMed, Scopus, Science Direct, ProQuest, Embase	
Limits: language: English; in title/abstract (keywords); full text available; document type: article, review, dissertation and thesis	
Publication date: 2000 up to 25 August 2020	
#1	“Evidence-Based Decision-Making” OR “Evidence-Based Management” OR “Evidence-Based Policy-Making” OR “Evidence-Informed Decision-Making” OR “Evidence-Informed Policy-making”
#2	Barrier* OR limit* OR inhibit* OR hinder* OR prevent* OR prohibit* OR obstacle* OR hurdle*
#3	Facilitate* OR accelerate* OR enable*
#4	Health* OR hospital*
Search strategy	1. #1 AND #2 AND #4
	2. #1 AND #3 AND #4
Example (Scopus database)	**1. ( TITLE-ABS-KEY (** ***“Evidence-Based Decision-Making”*** **OR** ***“Evidence-Based Management”*** **OR** ***“Evidence-Based Policy-Making”*** **OR** ***“Evidence-Informed Decision-Making”*** **OR** ***“Evidence-Informed Policy-making”*** **) AND TITLE-ABS-KEY (** barrier* **OR** limit* **OR** inhibit* **OR** hinder* **OR** prevent* **OR** prohibit* **OR** obstacle* **OR** hurdle* **) AND TITLE-ABS-KEY (** health* **OR** hospital* **) AND LANGUAGE (** ***english*** **) ) AND DOCTYPE (** ***ar*** **OR** ***re*** **) AND PUBYEAR >** ***2000***
	**2. ( TITLE-ABS-KEY (** ***“Evidence-Based Decision-Making”*** **OR** ***“Evidence-Based Management”*** **OR** ***“Evidence-Based Policy-Making”*** **OR** ***“Evidence-Informed Decision-Making”*** **OR** ***“Evidence-Informed Policy-making”*** **) AND TITLE-ABS-KEY (** facilitate* **OR** accelerate* **OR** enable* **) AND TITLE-ABS-KEY (** health* **OR** hospital* **) AND LANGUAGE (** ***english*** **) ) AND DOCTYPE (** ***ar*** **OR** ***re*** **) AND PUBYEAR >** ***2000***

### Selecting and refining the studies

The inclusion criteria consisted of those articles in any formats of review, original articles, or dissertations with the English language that had a full text and was determined or identified facilitators or barriers of EBM in health systems. Also, the exclusion criteria were the studies without full text or English language and some types of articles like a book review, opinion articles, or commentaries that had no defined framework for inspecting this study’s intended factors. In addition, the studies that were conducted clinically regarding the various scopes of health, medicine, or diseases were excluded. During this step, it was attempted to inquire about the related gray literature or studies that were not included in the search process as far as possible by reviewing the reference lists of the selected studies or by contacting some experts or the authors of the articles. This complementary search was based on the related articles’ titles in the reference lists of the selected articles conducted in Google scholar.

**Fig. 1:**
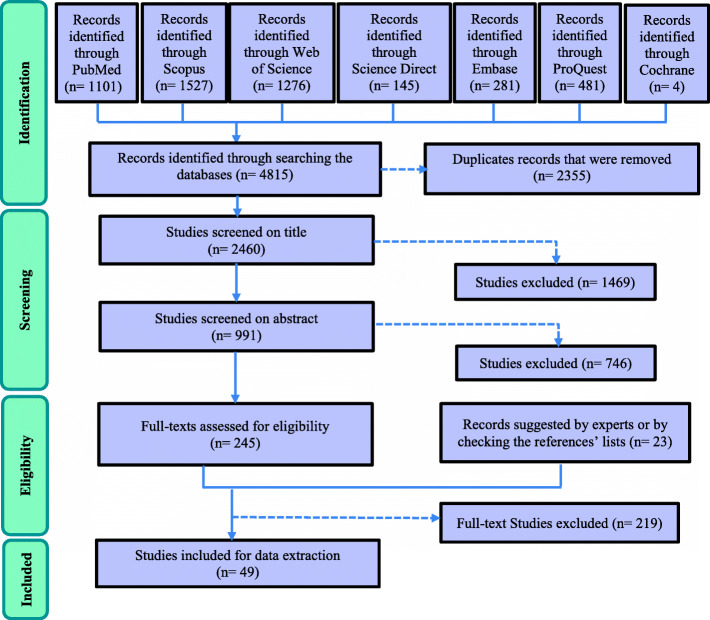
The PRISMA flow diagram for articles’ selection

After searching the studies from all databases and eliminating duplicates, the studies were independently reviewed and screened by two members of the research team (TSH and MRAM) in three phases by title, abstract, and then the full text of the articles. At each phase, the final decision to include the evidence was based on agreement, and in case of disagreement, the opinion of the third member (PB) was used. The Mendeley software (version 1.19.4) was used to manage the process of systematic search. At the same time, the Preferred Reporting Items for Systematic Reviews and Meta-Analyses-extension for Scoping Review (PRISMA-ScR) [20] was applied to manage the process of including the related evidence (see supplementary files 1 for PRISMA-ScR checklist and Fig. 1 for PRISMA flow Diagram). Since in the scoping review, appraising the quality of the searched studies is not obligatory [21], the quality of the obtained studies was not appraised via the standard guidelines. However, as it was clarified before, the third member (PB) finally screened full-text studies for eligibility, adhering to those same criteria and the relevance of the included studies aims. As it is obvious via this detailed process in the third step, we have both determine the approach of selecting the studies and excluding the data according to the third step of Levac et al. [19] and Arksey and O’Malley [18] as well.

### Analyzing and tabulating key information

This step is defined as charting the data in the protocol of Arksey and O’Malley and the incorporation of a numerical summary and the qualitative analysis via Levac et al. [19] approach. To cover both, in this step, after selecting the final studies based on the desired inclusion and exclusion criteria, data on the facilitators and barriers of EBM in health systems were extracted and included in data extraction forms applying Microsoft Excel 2013. The first author’s name, place, time of the publication, aim of the study, study design, and the study setting were included in the data extraction form. The results of this step are described in Table 2 in Additional file 2. At the same time, for better illustration of the evidence, the streamgraphs were drawn applying www.plotdb.com.

Then, for qualitative summative analysis, the included data was reviewed several times to be assured of considering all large or small sections of the included texts. The aim of the summative analysis is to cover all the complex subjects and contents of the text associated with the context [22]. Applying this method of analysis in this step helped us to develop and summarize the main aspects related to the facilitators and barriers of EBM and clarify the fundamental meaning of a text and its properties.

### Summarizing and reporting

At this step, two researchers (TSH and MKRZ) independently integrated and summarized the texts to reach the main and sub-aspects related to the facilitators and barriers of EBM. At the times of probable disagreements, the third person in the research team (PB) who has more reflexivity helped to reach the consensus. These aspects then were defined, clarified, and tabulated as a comprehensive set of all facilitators and barriers to EBM in health systems and organizations (Tables 3 and 4 in Additional file 2). The qualitative software MAXQDA version 10 was applied in this step. In this regard via the fifth step, the aim to identify the implications for practice and the policymakers as well was conducted via the evidence summative analysis. This is the point that Levac et al. [19] mentioned in their protocol.

### Consulting with the experts

This step was mentioned optional according to Arksey and O’Malley. Although the revised protocol by Levac et al. [19] emphasized that achieving the viewpoints of the experts via consultation can be a required and necessary component. For solving the conflict among the approaches and achieving an illustrative map of the facilitators and barriers of EBM, we have obtained the consultation of some of the national experts on the finalized tabulated results.

## Results

Based on a systematic search, 4815 studies were found from 7 databases, reaching a total of 2460 articles after duplicates were removed. After excluding studies with unrelated titles, 991 studies remained, and after studying the abstracts and removing unrelated articles, 268 articles were selected for the full-text screening. After studying the full text of the remaining papers, 49 papers were eventually selected to extract facilitators and barriers to EBM in health systems (Fig. 1).

Most of the studies are (22 (44%)) conducted between 2011 and 2015, 15 (30%) of them conducted between 2016 and 2020, 8 (16%) of them between 2006 and 2010, and the others (4 (8%)) conducted between 2000 and 2005. Additionally, most of the selected articles were from the USA (16 (32%)), Canada (11 (22%)), and Australia (6 (12%)). Also, there were 5 (10%) studies from Iran, and the rest (11 (22%)) were from other countries. Considering the design of the studies, 24 (48%) were qualitative, 18 (36%) were quantitative, and 7 (14%) were mixed-method researches. A summary of the final selected articles is given in Table 2 in Additional file 2.

Results of the summative analysis have shown that six main aspects attitude toward EBM, external factors, contextual factors, resources, policies and procedures, and research capacity and data availability were summarized as EBM facilitators. These six aspects were classified into 24 sub-aspects presented in Table 3 in Additional file 2 (see supplementary files 2 at the end of the text). Other results of the summative analysis have demonstrated that the barriers to EBM were similarly summarized as attitude toward EBM, external factors, contextual factors, policies and procedures, limited resources and research capacity, and data availability. These EBM barriers’ main aspects in health systems were also classified into 27 sub-aspects presented in the Table 4 in Additional file 2 (see supplementary files 2 at the end of the text). Definition and clarification of the concept achieved by the summative analysis have declared that factors that contributed to the development and implementation of EBM in the organization among the included texts were categorized as facilitators and factors that prevented or hindered the promotion of EBM in the organization among the retrieved texts were classified as barriers.

**Fig. 2:**
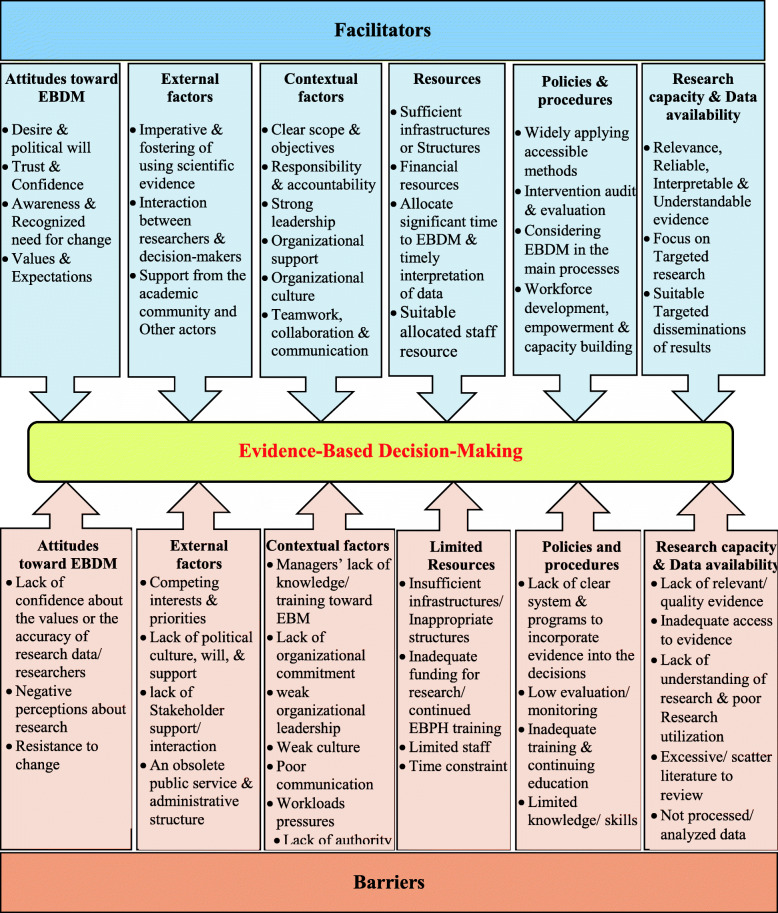
The framework of facilitators and barriers of EBM in health systems

**Fig. 3:**
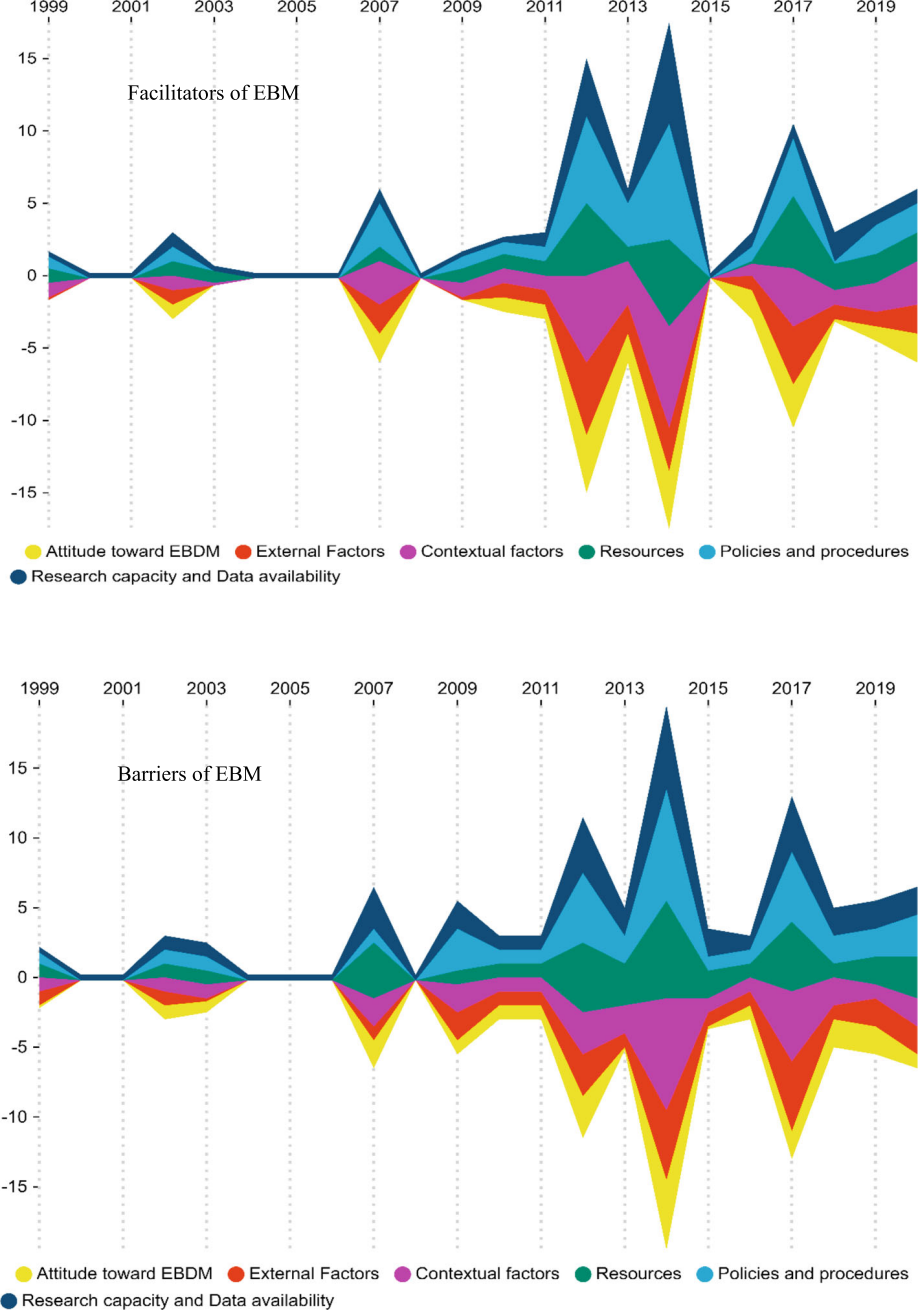
The general trend of the facilitators and barriers of EBM in health systems

**Fig. 4:**
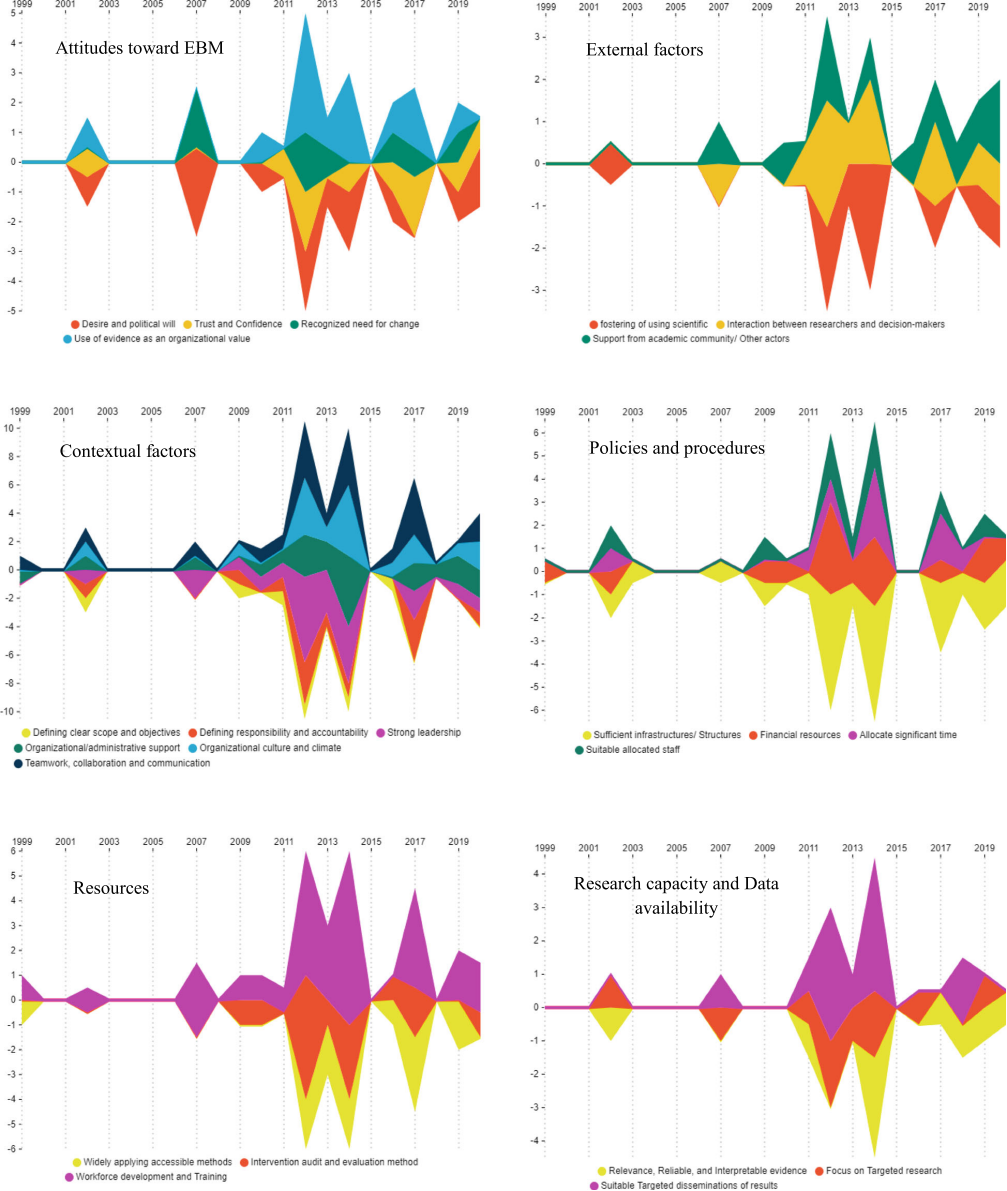
Comparison of the quantity and publication year of the retrieved studies according to the main aspects and sub-aspects of EBM facilitators

**Fig. 5:**
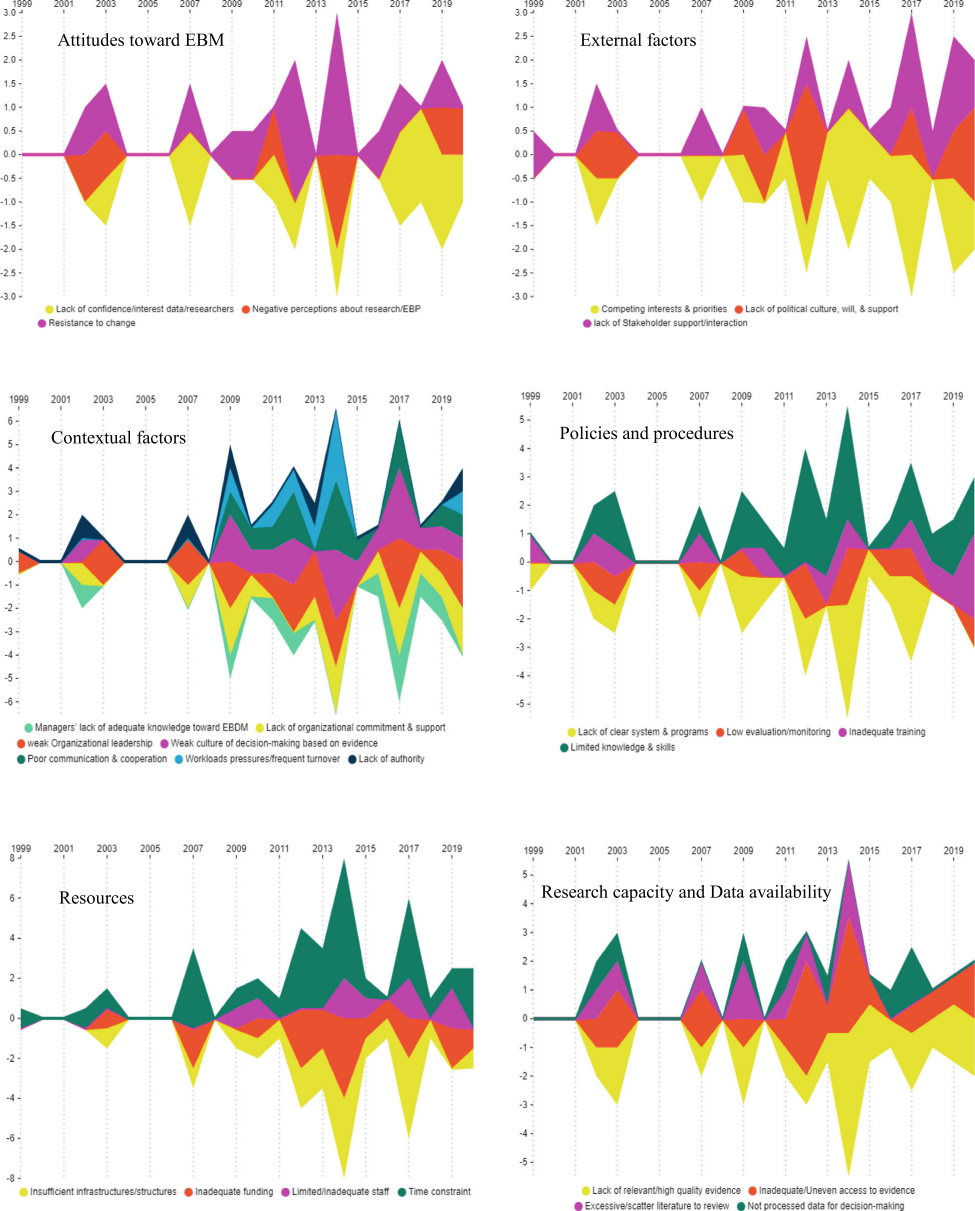
Comparison of the quantity and publication year of the retrieved studies according to the main aspects and sub-aspects of EBM barriers

Also, the framework of facilitators and barriers of EBM in health systems is illustrated in Fig. 2. Additionally, the general trend of facilitators and barriers of EBM in health systems and comparison of the quantity and publication year of the retrieved studies according to the main aspects and sub-aspects of EBM facilitators and barriers are illustrated in Figs. 3, 4, and 5, respectively. As it is obvious in these streamgraphs, the international attention to the sub-aspects of facilitators and barriers of EBM has been increased since 2011, and all the sub-aspects were included in different studies from that period.

## Discussion

In the present study, we provide a comprehensive map of the facilitators and barriers to EBM in health systems that have been classified into six main aspects including “attitudes toward EBP and research,” “external factors,” “contextual factors,” “policies and procedures,” “resources,” and “research capacity and data availability”. In a study, facilitators and barriers to evidence use in program management and decision-making within health care organizations were divided into four distinct groups: informational, organizational, individual, and interactional [10]. Another study revealed seven themes to describe both barriers and facilitators: training, attitudes, consumer demand, logistical considerations, institutional support, policy, and evidence [15].

In this regard, however, several studies were conducted to identify facilitators and barriers of EBM or EBDM in health organizations; they only focused on some aspects of just one or two of these factors and did not present a comprehensive and complete set or framework for them [11–16].

In the following, we discussed the main aspects in two general categories of facilitators and barriers to EBM.

### EBM facilitators

In this research, EBM facilitators were categorized into six main aspects and 24 sub-aspects. Humphries et al. divided facilitators into five principal themes (information, structure and process of the organization, culture of the organization, and individuals’ skills and interactions), and 15 sub-theme [10]. Jessani et al. mentioned nine domains for facilitators (financial, time, work culture, networks, experience, instructional reputation, geographic location, other actors, and relevance) [23]. Sosnowy et al. divided factors affecting EBDM into two main scopes: internal and external factors with themes such as strong leadership, workforce capacity, resources, funding and program mandates, political support, and access to data and program models suitable to community conditions [24]. In this regard, some of the differences in the categorization of the EBM or EBDM facilitators may be due to the type, scope, extent, and main objectives of the studies. Also, the different attitudes of the authors can lead to the various classification of the factors. However, none of the previous studies did present a complete and comprehensive classification of the factors that facilitate the development and implementation of EBM in the health system or had not examined the trend or recurrence of these affecting factors on EBM.

### Attitudes toward EBM

In the “attitudes toward EBM” aspects, four sub-aspects that were identified the most frequent ones based on the previous studies were “use of evidence as an organizational value” (14 (28.5%)) and “desire and political will” (13 (26.5%)). Schleiff et al. in their study explained that EBDM does not take place in a depoliticized vacuum. Political alliances and priorities, knowledge brokers, and other factors have a substantial role to play in applying EBM in health organizations. Hence, after the leaders determined the evidence priorities, they can identify processes for their generation and use them by using political commitments to set up structures to support it [17].

### External factors

In this aspect, the most mentioned sub-aspects were “interaction between researchers and decision-makers and participatory decision-making” (12 (24.5%)). This interaction assists to create consensus between researchers and managers or decision-makers, which can facilitate and promote evidence use [10, 14, 25–32]. The relationship between researchers and decision-makers leads to making decisions on more accurate, reliable, and up-to-date information and thereby avoid waste of limited resources. Building or strengthening partnerships with schools, hospitals, community and social services organizations, private businesses, universities, and law enforcement can increase EBM in organizations, too [30].

### Contextual factors

Among six sub-aspects of the “contextual factors” aspect, “strong leadership” (23 (47%)), “organizational/administrative support” (22 (45%)), and “teamwork, collaboration, and communication” (21 (43%)) were the most repeated concepts in the literature. Encouragement of decision-makers to use evidence in their decision-making process can be considered as a change in organizations. Strong leadership and organizational support are the crucial components of a successful change in any organization [33]. On the other hand, proactive leadership can be associated with a more positive attitude toward evidence-based practice (EBP) [34]. Provision of incentives and motivations [2, 10, 17, 27, 28, 30, 35, 36] and explicit effort to capture synergies between various components of the organizations [28] by a strong and determined leader are the actions that can encourage the members to focus more on the EBM. As well as, presence of multidisciplinary, diverse management teams [30], virtual communication networks [29, 32, 35], interactive web-based meeting (webinars) [35], face-to-face meetings [28, 30] and brainstorming [10, 29], and use of common language and terminology [30] can facilitate teamwork and consequently enhance the use of evidence in the decision-making process in the organizations.

### Resources

This aspect included four sub-aspects, and among them, “sufficient infrastructures or structures” was the most mentioned sub-aspect (24 (49%)) in the studies. In this regard, some factors such as information systems [2, 27, 28, 30, 35] and technical infrastructure [2, 14, 28, 37]; appropriate wireless, internet, and intranet access and computers [14, 16, 17, 28, 29]; digitization of datasets, reports, and processes [17]; access to research and library services [10, 12, 36, 38–42, 13, 14, 17, 25, 27, 30, 32, 35]; knowledge on management tools [25, 30, 38, 39]; and the existence of a department for quality assurance [28] can have a great impact on providing the necessary infrastructure for EBM and promoting it in the organization.

### Policies and procedures

This aspect included four sub-aspects. “Workforce development, empowerment and training leaders/staff” was the most frequent sub-aspect (31 (63%)) in this aspect. Empowering the decision-maker and building capacity to use evidence in the decision-making process can lead to more usage of evidence in an organization. Also, evaluating the implementation of the decisions taken can lead to reinforcing and institutionalizing the use of EBM in the organization. Considering this, some factors such as “executive training programs” [2, 27, 30, 39], “leadership training” [25, 30], “offering the organization as a learning laboratory for Ph.D. and other senior students” [43], “increasing number of graduate programs that incorporate training in empirically supported treatments” [15], “conduct interactive workshops” [28, 44], “consultations” [44], “sending staff to external training programs,” “adapting training to specific specialties or clienteles” [28], “in-service and multidisciplinary training,” and “skills-based training” [30] can improve EBM. Decision-maker needs to learn how to gather and appraise evidence [5]. Training the individuals about EBM may enrich their attitude and understanding of the importance of EBM [14].

### Research capacity and data availability

In this aspect, three sub-aspects have existed. Accordingly, “relevance, reliable, interpretable and understandable evidence” was the most mentioned sub-aspect (10 (20%)) in studies. Evidence is the fundamental part of EBM, so the data for use should be real-time, synthesized, and from different agencies [17], and if so, the managers can make good decisions. Without this information, wrong decisions will be made, and it can lead to not only the organization that does not improve but may push it away from its desired goals.

### EBM barriers

Different types of factors were explained which can impede the development of EBM in the organization. In this research, the identified barriers in literature are divided into six main aspects with 27 sub-aspects. Liang et al. identified 12 barriers in three levels including a broader level, organization, and individual manager [27]. Humphries et al. identified five main themes (information, the structure and process of the organization, the culture of the organization, and individuals’ skill and interaction) and 28 sub-themes [10]. Pagoto et al. identified six themes for barriers: attitude toward EBP, training, logistical, policy, evidence, institutional support, and consumer demand [15] which is somehow similar to this study. Majdzadeh et al. mentioned three main themes (decision-makers’ characteristics, decision-making environment, and research system) and 14 sub-themes for EBDM barriers in Iran’s health system [45]. Again, none of the previous studies about the barriers of the EBM in health systems did present a complete set of factors. Moreover, it seems that the type, scope, extent, and main objectives of the studies and also the different attitude of the authors leads to the various classification of the factors.

### Attitudes toward EBM

In this aspect, both the “resistance to change” (14 (28.5%)) and “lack of confidence/interest about the values or the accuracy of research data or the researchers” (13 (26.5%)) were most repeated in previous researches. Adaption to various changes in organizations is unavoidable [46]. Resistance to change may be due to inappropriate use of power, challenges to cultural norms and institutionalized practices, lack of understanding, inappropriate timing, inadequate resources, incorrect information, or employees’ suspicion of honorable management intentions [47]. Moving toward EBM is considered as a change in an organization that causes fear for the managers or staff. Fear of change toward the unknown leads to resistance to change, so proper strategies and policies such as training, education, or compensations are essential to successful changes [46].

### External factors

In the “external factors” aspect, “competing interests and priorities” which is defined as “the need for a hierarchy of approaches that allow to competing for organizational priorities and a balance between reactive and proactive management” [48] was most cited (17 (35%)) in the literature. It was explained in the studies that often centralized [49], heterogeneous [31], or politically influenced decisions [10, 50] might prevent the managers of the health organizations from making efficient decisions based on the best available evidence.

### Contextual factors

Among the “contextual factors,” “weak Organizational leadership” (20 (41%)) and “weak culture of decision-making based on evidence” (18 (37%)) were the most repeated sub-aspects in the literature. It is clear that no program or change in the organization will be successful without the commitment and support of the leader and senior officials of the organization. Also, the implementation of any plan and reforms requires the existence of a suitable cultural context and infrastructure. Culture is an important basic element to support changes in an organization, as well as to move toward EBM [51]. Organizational culture plays a significant role in innovation and changes [52]. Developing a dominant culture for EBM is essential in organizations to ensure that decisions are well appraised by research evidence.

### Policies and procedures

In this aspect, “limited knowledge and skills to access, interpret, appraise, and synthesize research evidence, or in research methods or foreign language” was the most cited sub-aspect (26 (53%)) by the previous studies. According to a previous study, inadequate technical training to enable managers to interpret research findings was a barrier to adequate accessibility to scientific evidence [53]. Applying EBM needs to learn how to search and evaluate different evidence critically from scientific findings to experts’ opinions and even some economic data, which requires some new managerial skills [5]. Besides, training the staff about EBDM can lead to not only an understanding of the importance of its implication in the organization but also they can learn how to acquire, assess, adapt, and apply researches in the organizational decision-making process [14]. Also, Walker et al. stated that librarians could be a crucial part of improving understanding and use of evidence in the organization by raising awareness of evidence-based resources among the employees. Thus, creating a strong communication between librarians and decision-makers can increase the use of evidence [54].

### Limited resources

“Time constraint for collecting and interpretation of information, engaging in research or implementation of an evidence-based decision making” was identified as an important and frequent (33 (67%)) sub-aspect in the “limited resources” aspect. Health workers are overworked, so time constraints are one of the barriers to using evidence. Organizations should provide the essential tools to facilitate quick and easy access to the required research, ensuring appropriate journal subscriptions, and providing relevant links on the organizations’ intranet [14] to overcome these time constraints to some extent.

### Research capacity and data availability

“Lack of relevant or high-quality evidence” (24 (49%)) and “inadequate/uneven access to evidence” (22 (45%)) were the sub-aspects that were mentioned in much other literature. Uncertain/unreliable evidence [40, 42], non-useful format [31], not available data in an extractable format [55], and gaps in evidence [24, 36, 41, 48]/inadequate research findings [16] were mentioned by other studies as the items that can prevent the evidence-based decisions. Limited access to the electronic databases and experts’ opinions leads to barriers in using evidence in the decision-making process [42]. Evidence is the main part of the EBDM process; therefore, inadequate access to evidence can make it difficult to go toward EBM.

## Conclusion

The importance of decision-making regarding complex health systems, especially in terms of resource constraints and uncertainty conditions, makes it necessary to apply the EBM in the health system organizations as much as possible. Existence and access to credible evidence from a variety of sources can reduce uncertainty and opinion-based decision-making. Therefore, we tried to provide a comprehensive map of EBM facilitators and barriers in health system organizations since we did not find a study that provided a comprehensive synthesis of all facilitating and hindering factors to EBM. We expect that the authorities and managers of health system organizations can make evidence-based decisions in their organizations using the map and the complete set of potential EBM facilitators and barriers presented in this study and by focusing on improving the facilitators and reducing or eliminating the barriers. Such systematic, reliable, and rational decisions can properly justify the stockholders’ demands and at the same time lead to better use of limited resources in the organizations.

### Strengths and limitations of the study

The most important strength of this study is providing a comprehensive set of EBM facilitators and barriers in health systems and map their trends over years. At the same time, the main novelty and contribution to the knowledge of the study is the integration of two methodologies for conducting the scoping review.

However, like any other review, in this scoping review, some relevant sources of information might have been omitted, and the review was dependent on information on the review question and the selected search strategy. Also, for further studies, it might be interesting to survey the factors provided in this study from the healthcare managers’ perspective in different contexts.

## Supplementary Information

**Additional file 1.** Preferred Reporting Items for Systematic reviews and Meta-Analyses extension for Scoping Reviews (PRISMA-ScR) Checklist.

**Additional file 2: Table 2.** Summary of characteristics of included studies. **Table 3.** The facilitators of EBM in health systems. **Table 4.** The barriers of EBM in health systems management.

## Data Availability

Data charting is available as an additional file.

## Abbreviations

EBM: Evidence-based management
EBDM: Evidence-based decision-making
EBP: Evidence-based practice
PRISMA: Preferred Reporting Items for Systematic Reviews and Meta-Analyses
PRISMA-ScR: Preferred Reporting Items for Systematic Reviews and Meta-Analyses- extension for Scoping Reviews
