# Supplementary material for: A mapping of facilitators and barriers to evidence-based management in health systems: a scoping review study
Source: Syst Rev. 2021 Jan 30;10:42. doi: 10.1186/s13643-021-01595-8 (PMC7847165; doi:10.1186/s13643-021-01595-8)
Supplement: Supplementary file 2 — Additional file 2: Table 2. Summary of characteristics of included studies. Table 3. The facilitators of EBM in health systems. Table 4. The barriers of EBM in health systems management. [file 13643_2021_1595_MOESM2_ESM.docx]

**Appendix**

Table 2: Summary of characteristics of included studies

|  | First Author (Year) | Country | Title or Aim of the Study | Study Design | Setting |
| --- | --- | --- | --- | --- | --- |
| 1 | Ali Janati (2018) | Iran | An Evidence-Based Framework for Evidence-Based Management in Healthcare Organizations: A Delphi Study | Mixed-method study; a systematic review, two-round Delphi survey  and a series of semi-structured interviews | in the first round=45,  in the second round=21 |
| 2 | Prue F. E. Addison (2016) | Australia and New Zealand | Conservation practitioners’ perspectives on decision triggers for evidence-based management | Qualitative study; workshop | 15 |
| 3 | Mohsen Saberi Isfeedvajani (2018) | Iran | Evidence-Based Management and its Application in the Hospital Management Process | ---- | ---- |
| 4 | April L. Wright  (2016) | Australia | Evidence-based Management in Practice: Opening up the Decision Process, Decision-maker and Context  Evidence-Based | Qualitative study; Case study/ interview | 24 emergency physicians and registrars, four hospital executives and one nurse |
| 5 | Damian Eisenghower Greaves (2017) | Caribbean island | Evidence-based management of Caribbean health systems: barriers and opportunities | Qualitative study; semi-structured interviews | 20 senior managers/leaders |
| 6 | Havva Arslan Yurumezoglu (2012) | Izmir, Turkey | Pilot study for evidence-based nursing management: Improving the levels of job satisfaction, organizational commitment, and intent to leave among nurses in Turkey | Quantitative study; quasi-experimental, pretest–post-test design, questionnaire | 58 staff nurses, 137 staff |
| 7 | Hayfaa A. Wahabi (2015) | Riyadh, Saudi Arabia | Evidence-Based Decision Making in Public Health: Capacity Building for Public Health Students at King Saud University in Riya | Quantitative study; questionnaire | 33 student |
| 8 | Ross C. Brownson (1999) | Missouri, US | Evidence-Based Decision Making in Public Health | Qualitative study; work-team and pilot testing during a four-day course | 12 mid-level manager in the Missouri department of health |
| 9 | Marguerite Pappaioanou (2003) | USA | Strengthening capacity in developing countries for evidence-based public health: the data for decision-making project | Quantitative study; experimental | Bolivia, Cameroon, Mexico, and the Philippines’ health system |
| 10 | Jennifer Yost (2014) | Ontario, Canada | Tools to support evidence-informed public health decision making | Qualitative study; a case study design | A purposive sample of senior management and public health professionals |
| 11 | Wesley S. Gibbert (2013) | US | training the workforce in EB public health: an evaluation of impact among the US and international practitioners | Mixed-method study; follow up survey | 15- question online survey/ all participants (n=626) in course |
| 12 | Ross C. Brownson (2014) | U.S | Understanding Administrative  Evidence-Based Practices Findings from a Survey of Local Health Department Leaders | Quantitative study; cross-sectional, questionnaire | 517 local health department directors |
| 13 | Paul R. Falzer (2009) | USA | A Conditional Model of Evidence-Based Decision Making | Qualitative study | 21 residents at one psychiatry training program |
| 14 | Zhanming Liang (2012) | Australia | A framework to improve evidence-informed decision-making in health service management | Quantitative study; review of academic literature | 46 studies were included |
| 15 | Li, Yan (2015) | New York, USA | Advancing the Use of Evidence-Based Decision-Making in Local Health Departments With Systems Science Methodologies | Qualitative study; Small group interviews and focus groups | Participants from 31 LHDs (Local health departments) who had decision-making responsibilities. |
| 16 | Humphries, Serena (2014) | Canada | Barriers and facilitators to evidence-use in program management: a systematic review of the literature | Quantitative study; systematic review  of the literature | 14 papers were included in the review |
| 17 | B. M. Niedzwiedzka (2003) | Poland | Barriers to evidence-based decision making among Polish healthcare managers | Quantitative study; questionnaire | 815 |
| 18 | Moriah E Ellen (2014) | Canadian provinces (Ontario and Quebec) | Barriers, facilitators, and views about next steps to implementing supports for evidence-informed decision-making in health systems: a qualitative study | Qualitative study; semi-structured telephone interviews | Fifty-seven managers |
| 19 | Leslea Peirson (2012) | Ontario, Canada | Building capacity for evidence-informed decision making in public health: a case study of organizational change | Qualitative study; case study, semi-structured interviews, and focus groups | 70 members of the health unit, and through a review of 137 documents |
| 20 | Julie A Jacobs (2014) | four U.S. states: Michigan, North Carolina, Ohio, and Washington | Capacity building for evidence-based decision making in local health departments: scaling up an effective training approach | Quantitative study; Semi-experimental through a training course | 130 participants |
| 21 | Megan Ward (2012) | Ontario, Canada | Creating an organizational culture for evidence-informed decision making | ---- | ----- |
| 22 | Tekabe Belay (2009) | the states of Rajasthan, Maharashtra, and Uttar Pradesh, India | Data Utilization and Evidence-Based  Decision Making in the Health Sector | Quantitative study | 270 officials and health managers from the central, state, district and below district levels. |
| 23 | Nasreen Jessani  (2017) | Kenya | Enhancing evidence-informed decision making: strategies for engagement between public health faculty and policymakers in Kenya | Qualitative study; semi-structured interviews | academic KBs and university leaders from six Schools of Public Health (SPHs) as well as national policymakers |
| 24 | Mary McDiarmid (2007) | Ontario | Evidence-based administrative decision making and the Ontario hospital CEO: information needs, seeking behaviour, and access to sources | Qualitative study; telephone interview survey | 35 CEO |
| 25 | Melanie Kazman Kohn (2011) | ---- | Evidence-based decision making in health care setting: from theory to practice | ---- | ---- |
| 26 | Linda Weiss (2012) | New York, USA | Evidence-Based Decision Making in Local Health Departments | Qualitative study; Focus group, interviews | 47 LHD commissioners, health directors, and other upper-level staff |
| 27 | Shirley Wallington (2002) | Canada | Evidence-based decision making: An Integral Part of the System of knowledge | Qualitative study; an action research | 10 participants |
| 28 | Charment Oscar Moussata (2017) | Denver, USA | Evidence-based management and its influence on the practices of senior leaders of hospitals in the Denver metropolitan area | Exploratory qualitative study | 13 senior executive leaders of hospitals and health systems |
| 29 | Collette D. Sosnowy (2013) | New York, USA | Factors Affecting Evidence-Based Decision Making in Local Health Departments | Qualitative study; interview, focus groups | 20 individual |
| 30 | Ross C. Brownson (2012) | USA | Fostering More-Effective Public Health  by Identifying Administrative  Evidence-Based Practices | Quantitative study; a Review of the Literature (review of reviews) | synthesis of data from 20 reviews, identify a total of 11 high-priority EBPs |
| 31 | Sarah Bowen (2009) | Manitoba, Canada | More Than “Using Research”: The Real Challenges in Promoting Evidence-Informed Decision-Making | Qualitative study; focus group and interview | 17 focus groups and 53 semi-structured individual interviews involving 205 planners and decision-makers |
| 32 | François Champagne (2014) | Alberta, Saskatchewan, Quebec, and Nova Scotia | Organizational impact of evidence-informed decision making training initiatives: a case study comparison of two approaches | Mixed-method study | 84 people |
| 33 | Wilza Carla Spiri (2017) | São Paulo | Perception of Nursing Middle Managers about the Evidence-Based Management | Qualitative study; Case Study, individual structured interview | 15 middle managers |
| 34 | Ross C. Brownson (2014) | Washington University | Practice–Research Partnerships and Mentoring to Foster Evidence-Based Decision Making/ Domains, Definitions, and Examples of Evidence-Informed Mentoring for Practice-Based Research in Public Health | ---- | ---- |
| 35 | Maureen Dobbins (2007) | Ontario | Public Health Decision-Makers’ informational Needs and Preferences for Receiving Research Evidence/  To facilitate integration of research evidence into the decision-making process | Qualitative study; semi-structured interviews | 16 respondents public health decision-makers |
| 36 | Reza Majdzadeh (2012) | Iran | Strengthening evidence-based decision-making: is it possible without improving health system stewardship | Qualitative study; in-depth  interviews and focus group | Thirteen in-depth interviews and six FGDs |
| 37 | Claire Harris (2017) | Australia | Sustainability in Health care by Allocating Resources Effectively (SHARE) 7: supporting staff in evidence-based decision-making, implementation and evaluation in a local healthcare setting | Mixed-method study; literature reviews, surveys, interviews, consultation, and workshops | ---- |
| 38 | Claire Harris (2018) | Australia | Sustainability in Health care by Allocating Resources Effectively (SHARE) 8: developing, implementing and evaluating an evidence dissemination service in a local healthcare setting | Mixed-methods study; literature review, surveys, interviews, workshops, audits, document analysis, and action research | ---- |
| 39 | Rebekah R Jacob (2014) | U.S. | Training needs and supports for evidence-based decision making among the public health workforce in the United States | Quantitative study; questionnaires | 4 groups of local health department directors (first= 441, second= 904, third= 517, fourth= 332) |
| 40 | Rebecca Armstrong (2014) | Victoria, Australia | Understanding evidence: a statewide survey to explore evidence-informed public health decision-making in a local government setting | Mixed-method study; questionnaire, interviews | 135 responses were received and 13 interviews |
| 41 | Elizabeth A. Dodson (2010) (13) | State health departments | Use of Evidence-Based Interventions in State Health Departments: A Qualitative Assessment of Barriers and Solutions | Quantitative study; questionnaire | 469 |
| 42 | Moriah E Ellen (2013) | two Canadian provinces (i.e., Ontario and Quebec) | What supports do health system organizations have in place to facilitate evidence-informed decision-making? a qualitative study | Qualitative study; in-depth semi-structured telephone interviews | 57 interviews were conducted in 25 hospitals |
| 43 | Sherry L. Pagoto (2007) | USA | Barriers and Facilitators of Evidence-Based Practice Perceived by Behavioral Science Health professionals | Qualitative study; | 37 professionals |
| 44 | Amy Vratny (2007) | USA | A Conceptual Model for Growing Evidence-based Practice | Qualitative study; Case study | a licensed 200-bed, private, nonprofit regional referral hospital |
| 45 | Meike J. Schleiff (2020) | nine countries (Chile, Ethiopia, Ghana, Kyrgyzstan, Lebanon, Mozambique, Rwanda, South Africa, and Sri Lanka) | Comparative analysis of country-level enablers, barriers and recommendations to strengthen institutional capacity for evidence uptake in decision-making | Qualitative case studies. framework analysis comparing | Teams from nine countries |
| 46 | Ali Ayoubian (2020) | Iran | Evaluation of facilitators and barriers to implementing evidence-based practice in the health services: A systematic review | systematic review | 12 articles |
| 47 | Rose N. Oronje (2019) | Kenya & Malawi | Strengthening capacity to use research evidence in health sector policy-making: experience from Kenya and Malawi | cross-sectional descriptive studies that used both quantitative and qualitative methods | the project implemented a training and mentorship program for 60 mid-level policy-makers in the two MoHs |
| 48 | Nadeen Hilal (2020) | Lebanon | The use of evidence in decision making by hospital managers in Lebanon: A cross-sectional study | cross-sectional design based on a self-administered web survey | Middle and senior managers working in hospitals. |
| 49 | Edris Hasanpoor (2019) | Iran | Nursing Managers’ Perspectives on the Facilitators and Barriers to Implementation of Evidence- Based Management | cross-sectional study design | 276 nursing managers |

**Table 3: The facilitators of** EBM **in health systems**

| Themes | Sub-themes | | Related codes |
| --- | --- | --- | --- |
| Attitudes toward EBDM | Desire and political will (4,6, 16, 18, 19, 29, 27, 41, 43, 44, 45, 46,49) | Government support, Prioritize issues | |
|  |  |  | |
|  | Trust and Confidence (4, 14, 19, 25, 27, 28, 33, 40,46,49) | Interest and willingness to scientific decision-making/management principles | |
|  | Awareness/Recognized need for change (4, 19, 28, 30, 42, 43, 44,49) |  | |
|  | Use of evidence as an organizational value (2, 4,14,18,16,19,23, 27, 30,32,33, 41,42, 49) | Institutional reputation; Rationality; Excellence, innovation, and Flexibility; Fair in the decision-making process; Promotion of evidence and research utilization; Nonhierarchical decision-making and Shared employee perceptions | |
| External factors | Imperative and fostering of using scientific evidence (5, 16, 18, 26,27,29,30, 39,45,49) | Needs to be a Priority; Grant and regulatory requirements; National research agendas/ policies; National meetings/ mandates | |
|  | Interaction between researchers and decision-makers and participatory decision-making (14, 16, 18, 21, 25, 28, 30, 33, 35, 42,45,49) | Enhance partnerships with other related organization; Share data between civil servant & researchers/different agencies & sectors with government | |
|  | Support from the academic community/ Other actors (2, 3, 19,23, 30,39,41,43,45,46, 49) | Technical assistance; Learning to work with state legislatures; Making a connection with development partners; Collaboration between hospitals and schools; Use of management consultants | |
| Contextual factors | Defining clear scope and objectives (4, 22, 25, 27, 30, 32) |  | |
|  | Defining responsibility and accountability (5, 19,21, 22,25, 27, 28, 30, 33,39, 42,45) | Put EBDM in job descriptions; Seniors’ accountability | |
|  | Strong leadership (6,10, 11, 14, 16, 19, 21,22, 25, 26, 27, 28, 29, 30, 32, 33, 39,41,42,43, 44,45,49) | Provision of incentives and motivations; Alignment and explicit effort to capture synergies between various components | |
|  | Organizational/ administrative support (8,10,11, 14, 16,18, 21, 25, 27, 28,29, 30, 32, 33, 38, 39,41, 43, 46, 47,48,49) | Seniors’ Commitment and Involvement | |
|  | Organizational culture and climate (4, 10, 14, 16, 18, 19, 22, 23, 26, 27, 28, 30, 32, 40, 42,45,48,49) |  | |
|  | Teamwork, collaboration and communication (4, 5, 6, 8, 16, 19, 21, 23, 25, 27, 28, 30, 32, 33, 34,35, 39, 41, 42, 45,46) | Presence of multidisciplinary, diverse management teams; Networking with higher-level staff; Virtual communication networks; Interactive web-based meeting (Webinars); Use of social networks; Face-to-face meetings and Brainstorming; Use of Common language and terminology | |
| Resources | Sufficient infrastructures/ Structures (10, 14, 16, 17, 18, 19, 21, 22, 25, 27, 28, 30,33, 35, 36,37, 38, 39, 40, 42, 45, 46, 47, 49) | Information systems and technical infrastructure; Appropriate wireless, internet, and intranet access and computers; Digitization of datasets, reports, and processes; Access to research and library services; knowledge management tools; Existence of a department for quality assurance or research support | |
|  | Financial resources (8, 10, 14, 19, 21, 22, 23, 27, 30, 39, 40, 41, 42,45,47,49) | Use RBF (results-based financing); Funding projects through partnerships; Program financial risk | |
|  | Allocate significant time to EIDM and timely interpretation of data (3, 5, 10, 19, 23, 25, 27, 39, 40) |  | |
|  | Suitable allocated staff resource (19, 22, 27, 28, 30, 32, 42, 39,47) |  | |
| Policies and procedures | Widely applying accessible methods/strategies (2, 5, 8, 18, 19, 23, 29, 30, 33, 34, 42, 47, 49) | evidence-based programming suitable to local conditions; Utilizing decision support tools such as templates for the policy or procedure development; Establish flows to reach the proposed goals; Developing Action plans and multicomponent, active strategies; Using Conceptual models and adherence to the causal framework | |
|  | Intervention audit and evaluation method (2, 14, 16, 18, 19, 21, 22, 26, 28, 30, 33, 39, 41, 42, 45) | Use of economic evaluation; Tools and guidelines for monitoring and evaluation planning | |
|  | Considering EBDM in the main organizational processes (30, 33, 42, 46) | Recruitment and retention strategies; Place EBDM as a component of organization accreditation | |
|  | Workforce development, empowerment and Training staff/ leaders (5, 6, 8, 10, 11, 14, 16, 18, 19, 20, 21, 22, 23, 24, 25, 27, 28, 29, 30, 32, 33, 39, 40, 41, 42, 43, 44, 45, 46, 47, 49) | Staff with appropriate knowledge, skills, experience, ability to gathering data, and Critical thinking; Development of specific positions, such as Knowledge Brokers (KBs) or contracts with external KBs | |
| Research capacity and Data availability | Relevance, Reliable, Interpretable and Understandable evidence (16, 23, 25, 27, 32, 38, 40, 45,49) | Real-time data; Synthesized data; Data from different agencies | |
|  | Focus on Targeted research (2, 10, 14, 18, 19, 25, 27, 35, 42, 49) | Execute high priority research; Use of the systematic review | |
|  | Suitable targeted disseminations of results (3, 14, 16, 19, 21, 25, 30, 32, 34, 35, 38, 39, 42) | Attention to target user; Gathering electronic mailing lists to disseminate local research results; Providing research presentations both within and outside the organization; Provide or participate in presentations/ online/ or face-to-face briefings about specific reviews or review-derived products; Providing the executive summary or Policy briefs; Posting every rapid review on the externally facing library web site, along with a short video clip of the authors | |

* The numbers in parentheses are the reference numbers of the articles used to extract the EBM facilitators according to order of the studies that are placed in table 2.

Table 4: The barriers of EBM in health systems management

| Themes | Sub-themes | Related codes |
| --- | --- | --- |
| Attitudes toward EBP and research | Lack of confidence/interest about the values or the accuracy of research data or the researchers (3, 9, 16, 25, 28, 36, 37, 38, 43, 44,46,47,49) |  |
|  | Negative perceptions about research/EBP or toward change (16,17, 18, 25, 27,46,49) |  |
|  | Resistance to change (2, 9, 10, 14, 16, 26, 27, 31, 34, 36, 37, 41, 43, 49) | Fear of change or social-economic consequences; The need for rapid decisions; The impact of undesirable past experiences |
| External factors | Competing interests and priorities (2, 12, 15, 16, 25, 27, 28, 29, 31, 33, 36, 37, 39, 43, 45,47,49) | Difficulties in prioritizing local public health challenges and identifying process and outcome measures; Politically influenced decisions; Heterogeneous decisions; Centralized decision-making; Allocating resources to choices that are not related to national priorities |
|  | Lack of political culture, will, and support (5, 9, 14, 26, 27, 31, 36, 41,45,46, 49) | lack of understanding about using EBDM; The health ministry’s lack of co-operation in giving researchers the information they require; Absence of a common language between the health ministry’s policy-makers and the researchers; Poor Capacity of policy implementation environment/ lack of regulation and policy |
|  | lack of Stakeholder support or interaction (2, 3, 5, 8, 16, 23, 27, 36, 41, 43, 45, 47, 49) | Lack of external pressure; Researchers’ and policy-makers’ lack of awareness of each other’s abilities and needs; Incoordination and miscommunication between data units & policy-makers; Lack of communication between knowledge producer and organization decision-makers |
|  | Limitation of public services and administrative structures (5, 10, 22, 27, 41, 46) | The social, political, and historical context of public health practice; Frequent public health crises; Challenge of political, structural, and management issues; MOH Ministry Demands/Pressures; Government decisions which not based on information; Subjective norms |
| Contextual factors | Managers’ lack of adequate knowledge or training toward EBM (2, 3, 25, 27, 31, 33, 36, 37,49) | Lack of awareness of researchers’ ability and beliefs about capabilities; Lack of a vision or focus |
|  | Lack of organizational commitment and support (2, 11, 12, 16, 22, 27, 28, 31, 37, 38, 41, 43,45,46, 49) |  |
|  | weak Organizational leadership (8, 9, 11, 14, 16, 17, 22, 25, 28, 29, 31, 33 34, 36, 37, 43, 44, 45, 46, 49) | Lack of incentives and poor motivation; weak agenda setting; Weak governance skills; Lack of appreciation for the research process; More focus on care delivery/political processes rather than evidence use |
|  | Weak culture of decision-making based on evidence (2, 3, 5, 7, 10, 14, 16, 22, 23, 25, 27, 28, 31, 36, 40, 41, 46,49) | Challenging the promotion of evidence use; lack of crisis management culture; Lack of flexibility; Rules-oriented culture; |
|  | Poor communication and cooperation (7, 10, 16, 19, 23, 25, 31, 36, 37, 39, 41, 45,49) | Meetings and communication pathways |
|  | Workloads pressures (10, 27, 31, 16, 29) or frequent turnover (18, 25,45) |  |
|  | Lack of authority for decision-making/change practice (27, 29, 31, 44, 46) | Unfilled job openings |
| Policies and procedures | Lack of clear system and programs to incorporate evidence into the decisions (2, 5, 7, 8, 9, 13, 14, 16, 26,27, 28, 31, 32, 33, 34, 39, 41, 43) | Complexity or lack of organizational decision-making process and methods; Unclear definition of evidence and Confusing the definition of evidence-based; Lack of evidence-based models; Not integrating evidence from the public and private sectors; Failure to package and present data in an understandable and compelling format; Lack of information on the effectiveness of programs or certain interventions; Lack of action planning and adaptation intervention |
|  | Low evaluation or monitoring (2, 17, 22, 26, 27, 36, 37, 39, 40, 43, 46) | Not clear or suitable Indicators for performance; Lack of practice guidelines; Lack of consistent standards in the collection, production, and dissemination of data or choosing decision-makers |
|  | Inadequate training and continuing education (1, 8, 17, 27, 29, 33, 34, 41, 43, 45, 46, 48, 49) | Inadequate training in research methods |
|  | Limited knowledge and skills to access, interpret, appraise, and synthesize research evidence, or in research methods or foreign language (2, 9, 10, 13, 14, 16, 17, 18, 19, 21, 22, 23, 25, 26, 27, 29, 37, 38, 40, 41, 42, 43,45,46,47,49) | Insufficient capacity building; Limited opportunities for professional development/ educational and research restrictions; Lack of positions/roles for staff participation in evidence generation/use; Lack of knowledge in the application of training in real policy-makings |
| Limited resources (45,46) | Insufficient infrastructures/ Inappropriate organizational structure (2, 5, 10, 15, 16, 17, 18, 24, 25, 26, 28, 29, 31, 32, 33, 36, 37, 38, 41, 42, 45) | Inability to allocated resources; Insufficient journal subscriptions; Slow download speeds; Locks placed on websites; Lack of appropriate and relevant service on the internet |
|  | Inadequate funding to support the generation/use of research evidence or continued EBPH training (2,5, 7, 11, 12, 14, 16, 17, 18, 20, 23, 24, 26, 29, 36, 41, 43,47,48,49) | Lack of EBM’s influence on budget allocation |
|  | Limited/inadequate staff (5, 7, 16, 18, 31, 33, 41, 47,49) |  |
|  | Time constraint for collecting and interpretation of information, engaging in research or implementation of an evidence based decision making (5, 8, 10, 11, 12, 14, 15, 16, 17, 18, 19, 20, 21, 23, 24, 25, 26, 27, 29, 31, 33, 35, 37, 38, 40, 41, 42, 43, 44,45,46,47,48) |  |
| Research capacity and Data availability | Lack of relevant or high quality evidence (2, 3, 7, 9, 10, 12, 14, 15, 16, 17, 22, 23, 24, 25, 27, 29, 31, 32, 37, 40, 45, 46, 47, 49) | Uncertain/ Unreliable evidence; Non-useful format; Not available data in an extractable format; Lack of Comparable Data; Gaps in evidence/ inadequate research findings; Probabilistic, observational, seemingly inconclusive nature of epidemiologic data; Proprietary nature of data and costliness; Non-functional databases and inefficient health information systems; Non-prioritized researches |
|  | Inadequate/ Uneven access to evidence (9, 10, 14, 15, 16, 17, 19, 21, 24, 25, 26, 27, 29, 31, 32, 37, 38, 40, 44,45,46,47) | Inappropriate processes/ nonexistent permissions to access to required data; Lack of mechanisms or no repository for accessing research evidence |
|  | Lack of understanding of research and poor Research utilization (15, 16, 25, 26, 27, 31, 37, 41, 46) | Difficult to interpret and translate the latest evidence at the local level; Lack of capacity to conduct literature searches; Inadequate knowledge of sources; Results difficult to convey retrospectively; Mismatch of research to complex reality; Poor assessing the reliability, quality, relevance, and applicability of research evidence; Poor messenger choice or non-actionable messages; Difficulties with feedback; Poor dissemination of evidence |
|  | Excessive or scatter literature to review (14, 16, 17, 22, 25, 27, 31, 35, 40) |  |
|  | Not processed and not analyzed data for decision making (2, 5, 9, 11, 22, 25, 27, 28) | Lack of Analyst Support; Misuse of data; Unwillingness to accept shortcomings in data; Lack the technical guidance; The hesitancy of epidemiologists to aid in interpreting findings; Working with coworkers who do not have evidence-based public health training |

* The numbers in parentheses are the reference numbers of the articles used to extract the EBM barriers according to the order of the studies that are placed in table 2.
